# “Honorable Toward Your Whole Self”: Experiences of the Body in Fatigued Breast Cancer Survivors

**DOI:** 10.3389/fpsyg.2020.01502

**Published:** 2020-07-03

**Authors:** Cooper Penner, Chloe Zimmerman, Lisa Conboy, Ted Kaptchuk, Catherine Kerr

**Affiliations:** ^1^Department of Neuroscience, Brown University, Providence, RI, United States; ^2^Department of Neurosurgery, Cedars Sinai Medical Center, Los Angeles, CA, United States; ^3^Warren Alpert Medical School, Brown University, Providence, RI, United States; ^4^Beth Israel Deaconess Medical Center, Harvard Medical School, Boston, MA, United States; ^5^New England School of Acupuncture, Newton, MA, United States; ^6^Department of Contemplative Studies, Brown University, Providence, RI, United States

**Keywords:** theories of embodiment, interoceptive awareness, cancer related fatigue, patient centered care, team based qualitative analysis

## Abstract

**Introduction:**

Cancer Related Fatigue (CRF) is one of the most common and detrimental side effects of cancer treatment. Despite its increasing prevalence and severity CRF remains dismissed by the majority of clinicians. One reason for the apparent gap between clinical need and clinical undertaking is the penchant toward reductionist accounts of the disorder: a tendency to discount the interface between the lived experience of sufferers and the multi-dimensional etiology of CRF as it manifests adversely on a day-to-day basis.

**Methods:**

In order to better understand the interplay between social, bodily, and emotional components of the disorder we undertook semi-structured interviews with thirteen Breast Cancer survivors suffering from CRF, and then subsequently analyzed their responses using Team Based Qualitative Analysis.

**Results:**

Our analysis revealed multiple dimensions of the social and bodily underpinnings of fatigue. Most relevantly we found a consistent change in the level and quality of attention to bodily signals. This shift in awareness appeared to be directly connected to the experience of CRF and a newfound, “respect,” for the needs of the body. Furthermore, we found that many of the practices that were described as helpful in alleviating fatigue were oriented around eliciting a sense of embodied awareness, examples being: dance, yoga, and shamanic ritual. This relationship with bodily sensations existed in conjunction with the anxiety and trauma that arose as a result of cancer treatment.

**Conclusion:**

Our analysis suggests that the quality of awareness and relationship to bodily experience in CRF is a functionally relevant component of the disorder and should be considered as an experiential target moving forward.

## Introduction

### Background

Cancer-related fatigue (CRF) commonly accompanies Cancer and has been defined as, “a distressing, persistent, subjective sense of physical, emotional and/or cognitive tiredness or exhaustion related to cancer or cancer treatment that is not proportional to recent activity and interferes with usual functioning” (Berger et al., 2015). It is now estimated that 39% of individuals will be diagnosed with some form of cancer over the course of their lifetime (Howlader et al., 2016). For many of these persons the burden of disease will not end after treatment, but will continue to manifest as a host of factors that impair quality of life (QOL), and catalyze subsequent pathologies (Curt et al., 2000; Holley, 2000; Kurzrock, 2001; Schultz et al., 2003; Yabroff et al., 2004; Mustian et al., 2007; Roscoe et al., 2007; Bower, 2008; Brown and Kroenke, 2009; Saligan et al., 2015; Antoni et al., 2016). More so than depression, pain, or nausea, patients rank CRF as the post-treatment symptom that causes the most distress and interference in daily life (Young and White, 2006; Stone and Minton, 2008). CRF is reported by patients to be a sudden and debilitating loss of energy that is more severe, more persistent, and more incapacitating than any form of pre-cancer fatigue (Holley, 2000; Wu and McSweeney, 2007; Bower, 2014). For many individuals in the throes of CRF, resting, at best, grants only a momentary feeling of relief (Medysky et al., 2017). Given its severity many patients find CRF to be nearly impossible to articulate, making the disorder both detrimental to an affected individual’s QOL and difficult to characterize in clinically tractable terms (Wu and McSweeney, 2007; Duijts et al., 2014). To date, it has been proposed that CRF is derivative of anemia, hypothyroidism, cortisol dysfunction, serotonin deregulation, and a pathological inflammatory profile (Ryan et al., 2007; Dantzer et al., 2008; Bower, 2019). Though the last of these possibilities has garnered the most empirical evidence there remains no clear consensus on the pathological underpinnings of CRF.

Currently, despite the steadily increasing number of cancer survivors, as well as the exponential growth in Psycho-oncological research, CRF remains largely undiagnosed and undiscussed by clinicians (Koornstra et al., 2014). As one of this study’s participants put it, cancer-related fatigue is, “a silent cry” (Participant 10).

Models of CRF generally find their theoretical bearings in the context of the Biopsychosocial model of fatigue (White, 2004; Bower, 2014). This model emphasizes the role of psychological and sociological risk factors in exacerbating the initial physiological insult presented by cancer and the requisite treatments. Though this perspective has been instrumental in expanding earlier, purely biological, perspectives, it has also served in some cases to depreciate the severity of fatigue disorders and germinated the widespread belief that sufferer of chronic fatigue are able to overcome their symptoms through willpower alone (Geraghty and Esmail, 2016). For example, Wu et al., in a highly cited paper on the topic of CRF, characterize fatigued cancer survivors as either *fighters:* “Some people exercise their will to master their fatigued body and retain the sense of integrity and the feeling of not being controlled by the illness,” or conversely as having a tendency to *surrender*: “Some people may simply give up control when the body is unable to meet the demands or expectations” (Wu and McSweeney, 2007). The underlying assumptions of this war-oriented dichotomy are manifold. Most notable is the notion that one can through enough effort “master” the fatigued body as opposed to “simply giv(ing) up.” Susan Sontag writes in *Illness as Metaphor*, “Theories that diseases are caused by mental states and can be cured by will power are always an index of how much is not understood about a disease” (Sontag, 2001). Indeed, though CRF is singular in its consistent severity as well as the circumstances under which it arises, the experiences of patient’s suffering from CRF is paralleled by the reports of individuals suffering from a variety of other medically unexplained syndromes, examples being: the detrimental lack of understanding on the part of interpersonal relationships and clinicians (Åsbring and Närvänen, 2002; Epstein et al., 2006; Dickson et al., 2007; Bunzli et al., 2013; Band et al., 2015), the indescribable nature of living with a chronic unexplained illness (Nettleton, 2006; Dickson et al., 2008; Toye and Barker, 2010; Dow et al., 2012) anxiety about bodily sensations (Nimnuan et al., 2001; Schur et al., 2007), and a shifted level of attention toward bodily sensations (Witthöft et al., 2012; Van den Bergh et al., 2017).

Through asking participants to describe their bodily experiences of fatigue- how CRF feels and how they react to those feelings- we hoped to rigorously elucidate trends in the multi- dimensional and highly heterogeneous experience of CRF. Moreover we aimed to critically evaluate existing trends in the literature, notably the aforementioned notion that fatigue can and should be overcome through willpower. Our study’s participants provide important insights into what the experiential components of the disorder are, and perhaps even more importantly what they are not.

## Materials and Methods

### Participants

Participants (*n* = 13) were recruited through posters hung throughout Providence Rhode Island as well as snowball sampling. After contacting one of our research staff a set of questions were administered to assess the putative participant’s eligibility. In order to be eligible for the study participants had to have:

(1) Undergone treatment for Breast Cancer.
(2) Be at least 6 months out of active treatment.
(3) Rate their fatigue at least a three out of ten on a standard Likert scale.
(4) Be capable of speaking English.
(5) Be under 80 years of age.
(6) Have not been hospitalized for a psychiatric disorder at any point in the last year.

When the Likert Scale was administered participant’s were explicitly asked to, “Rate your fatigue over the course of the last week from one to ten, with ten being such immense fatigue that no movement whatsoever was possible and one being no fatigue at all.” All participants were informed of the study’s purpose prior to answering any questions.

*N* = 20 people contacted us with interest in the study, of these, four were ineligible because they rated their fatigue as being too low, and three initially agreed to give an interview and then subsequently dropped out. All Participants knowingly signed a consent form that was approved by Brown University Institutional Review Board.

### Interviews

Following pre-screening, (CP) met participants (*n* = 13) for a semi-structured interview (Barriball and While, 1994). Interviews lasted between thirty and two hundred and forty minutes, sometimes over the course of two meetings. If it seemed that a participant had begun to discuss a dimension of their experience that wasn’t explicitly prompted by one of the fourteen pre-scripted questions (see Table 1), CP encouraged them to discuss it in more detail. In this way, although all interviews started with the same basic set of inquiries they were allowed to develop organically, and often covered a diverse array of topics. CP then manually transcribed interviews. Twenty-six hours of interviews were collected, transcribed, and coded in total.

**TABLE 1:** Relevant demographics of included participants.

Participant number	Reported occupation	Surgeries received	Cancer treatments received	Years since last treatment at time of interview
1	Unemployed	None reported	Four rounds of chemotherapy	3
2	Retired medical librarian	Left Quadrantectomy Reconstructive Surgery	Chemotherapy	4
3	Equal employment opportunity investigator	Bi lateral Mastectomy Reconstructive Surgery Oophorectomy Hysterectomy	Chemotherapy and radiation	4
4	Reiki healer	Lumpectomy	Chemotherapy and radiation	2
5	Clinical psychologist	Lumpectomy	Chemotherapy and radiation	6
6	Student	Bi Lateral Mastectomy Reconstructive Surgery	Chemotherapy and radiation	4
7	Retired university records keeper	None reported	Chemotherapy	20
8	Office worker	Lumpectomy	Chemotherapy	4
9	Office worker	Bi lateral Mastectomy Reconstructive Surgery Oophorectomy Hysterectomy	Experimental therapy	3
10	Gardener	Lateral Mastectomy	Chemotherapy and radiation	3
11	Homeopathic healer	Lumpectomy Hysterectomy	Chemotherapy	3
12	Retired nurse	None reported	Radiation chemotherapy	2
13	Organizer for patient’s advocacy group	Lateral mastectomy	Chemotherapy	4

*Note that all participants were Caucasian (see Study Limitations for a further discussion on participant characteristics).*

Interview questions were designed by authors CP, (CK) and (CZ) following a literature review on previous qualitative analyses of CRF. These questions were specifically designed to elicit a far-reaching conversation about CRF with a specific emphasis on changed experiences of the body.

We utilized an inductive thematic saturation scheme to define when content saturation had been reached (Saunders et al., 2018). This process involved discussions between authors CP, CK, and CZ without any formal Team Based qualitative analysis. When it seemed that new themes had ceased to emerge, we halted recruitment. All participants were given a twenty-dollar visa gift card as compensation for their time and energy.

### Interview Analysis

In this paper we utilized methods that were based on Kathleen Macqueen’s team-based qualitative analysis (Guest and MacQueen, 2008). Following the collection and transcription of the 13 interviews CP,CZ, and CK privately read through each transcript and created three separate codebooks based off of emergent themes. An iterative process followed wherein differences between the three codebooks were discussed and resolved (Kerr et al., 2011a, b). As a function of these discussions a final conglomerate codebook was created. This codebook contained explicit descriptions of each emergent theme.

CP then went through each participant’s transcript once again and grouped relevant pieces of each interview into its requisite sub-category. LC and TK were also consulted about the validity of the codebook after its completion and helped to distill relevant themes (see Tables 3A–C for an abridged version of the codebook sections presented in this manuscript).

## Results

### The Felt Experience of Cancer Related Fatigue

Fatigue appeared to exist on two broad temporal scales for our participants. Most viscerally, there was what Participant 10 termed, “energy crashes,” sudden bursts of overwhelming exhaustion that required the immediate cessation of whatever action was at hand. These crashes forced many of our participants to radically reshape their day-to-day lives in an effort to accommodate these precipitous losses in energy (Table 3A section 1).

In addition to these sudden drop offs there was also a more difficult to define chronic experience of imbalance, as if the basic constituents of everyday sensation had been altered. *“It’s just a heavy feeling it feels like my body is heavy. I have to push it through the day, through the atmosphere, like the atmosphere is heavy”* (Participant 4).

For many of our participants the experience of sudden precipitous energy loss and chronic “heaviness” existed simultaneously, serving at once to shackle attempts to return to a pre-cancer pace of life and dampening those experiences that remained energetically feasible. The source of this two-faced fatigue was often inexpressible for the individuals we spoke with. It existed more generally as a gap between what was expected of the body and what was physically possible.

*“So I’m in a rut and that’s how I feel about everything, my mind is in a rut, my body’s in a rut*… *I don’t feel comfortable in my own skin anymore, and I just have no energy, it’s a lose-lose situation”* (Participant 8).

### Interpersonal Influences: Clinical and Familial

Because chronic fatigue experiences appear on the surface to be akin to the fatigue of healthy life, interpersonal relationships can become difficult as those without CRF struggle to understand its additional burden. It is this unknowable and yet apparent ordinariness that serves, in many cases, to prevent the crippling nature of CRF to be meaningfully addressed or articulated by loved ones, medical practitioners, or even the sufferer of CRF themselves.

*“(My clinicians) dismiss (cancer related fatigue), I’m tired of telling them, so I just go for my annual physicals now, and I don’t even bother to complain, because they just treat me like it’s nothing…part of me thinks that maybe the Doctors are right”* (Participant 8).

In fact, for many of our participants, CRF had never been defined as a possible outcome of their cancer experience. As such, exposure to our study was the first time they learned that chronic energy loss was a normal side effect of cancer treatment instead of an anomalous personal reaction.

*“When I saw the little, you know the (advertisement for your study on Cancer Related Fatigue), I think there’s a part that makes me say, Oh this is a thing? I mean I sort of think of it as a character flaw [laughing]. Whereas if other people are experiencing it too*… *I think it makes me feel like less guilty about it somehow”* (Participant 1).

The chronic lack of information on CRF was compounded by cultural views regarding what an appropriate level of energy should be, as well as an articulated or implicit desire on the part of family and medical staff to move past the tumultuous experience of cancer and return to normalcy. As with Participant 1, this often led to an experience of guilt and sense of responsibility for the experienced fatigue.

*“I just feel like I feel so lazy, I can’t get up, I’m not getting anything done. You just feel like a bum*… *The Protestant work ethic. You just keep going. And with this, you can’t, you just can’t*” (Participant 10).

Guilt arose not only as a result of ineffective social and medical support systems, but also as an internalized adherence to what Participant 10 termed the “Protestant work ethic.” The language surrounding this ethic often took the form of a moral imperative, as if the experienced lack of energy was a personal decision or as Participant 1 termed it, “character flaw.” This ethical dimension in the experience of CRF accentuates the metrics often utilized by cancer survivors to express the experience of fatigue. For many, CRF was defined on the basis of what they could do rather than how they felt.

*“For years, I didn’t know that I was exhausted. I just knew I could only work 25 hours a week or I’d be screaming at my kids, I’d be screwing everything up, you know it’s only over time that I came to realize – so it’s– it’s a very hard thing to pin down”* (Participant 7).

This means of structuring the experience of CRF around a sense of productivity rather than internal state places the onus of fatigue on the survivor themselves instead of deleterious physiological factors. This sense of responsibility was at times reflected in the pejorative stance some participants took to the notion of being affected by fatigue at all. *“I think it’s a lot of mind over matter. I mean does everyone else complain about how exhausted they are and they can’t do anything? Put their big girl pants on. Man up!”* (Participant 9). A sense of responsibility over one’s fatigue is of course not only an abstract psychooncological construct. For many of our participants there was simply not enough room in their day-to-day lives for the chronic exhaustion they experienced post-treatment. Across participants, this lead to an experience of CRF as something that existed outside the realm of definition, but rather as an extension of prevailing, often untenable, social circumstances.

“*Coupled or tripled on top of financial issues, now you’ve got nothing, all these bills to pay, your husband just left you because you’re not a woman anymore. Your kids are upset. They don’t know how to deal. So they’re acting out or acting in. Everyone is a disaster and there is no net to catch this*… *I’ve got a special needs son that goes back and forth between the states, I’m trying to drive him. puking my guts up, I can’t go to work*… *So fatigue. Yeah. Yep. Definitely. Definitely*” (Participant 12).

Though there were, of course, a number of positive exceptions to the social trends we’ve outlined in this section (see Table 2), generally speaking, the felt experience of CRF was not so much shaped by embedded social relations as it was obscured by them. Often the lack of information patients received coupled with implicit or explicit presumptions on the nature of fatigue lead sufferers to feel guilty and responsible for their lack of energy. Furthermore, the means of describing CRF often reflected this view such that the most relevant metric for CRF was an individual’s productivity rather than their internal state.

**TABLE 2:** Semi structured interview questions utilized during every meeting.

Semi Structured Interview Questions
Though we’ve gone over the consent form, before we begin do you have any questions?
Is there anything specific about your experience of Fatigue you’d like to share with me?
Will you tell me the broad outline of your cancer experience starting with your diagnosis?
Do you talk about fatigue differently depending on who you’re discussing it with? If so, how would you describe it with a friend? Concerned family member? Clinician?
Is there a specific part of your body that you feel your fatigue affects more than others? If so what part? - What are the qualities of fatigue in that body part? Are these qualities different from fatigue you might feel elsewhere?
Does your fatigue ever move throughout your body?
Do you ever experience whole body fatigue? If so, is the quality of this fatigue different from singular body part fatigue? In what ways are they different?
Does the quality of your fatigue change depending on your environment?
How largely does fatigue factor into your planning for the day, if at all?
How many times a day, on average, would you say you need to rest? What does rest entail?
Do you think others understand this need?
Do you think fatigue has changed the way you function? If so, how?
When you feel fatigued what actions do you take?
Have you found anything that is particularly effective in decreasing your fatigue? if so what is it?
Is your current level of fatigue comparable to what you experienced pre treatment? If not how is it different?
When during the day is your fatigue the worst? Why do you think that’s the case?

**TABLE 3:** Relevant code definitions and supplementary quotations that align with the discussed themes. **(A)** Quotations relating to how cancer related fatigue manifests in the body both as an omnipresent new feature of experience, and a sudden energy crash. **(B)** Quotations relating to interpersonal relationships and social factors that influence the experience of cancer related fatigue. **(C)** Quotations relating to the reported shifts in bodily awareness that occurred as a function of fatigue, as well as specific practices that helped to ameliorate fatigue.

Table 3A. The Felt Experience of Cancer Related Fatigue	Code Definition: (1) Any text where a participant gives specific objective easily applicable information about the nature or location of their fatigue. (2) Any text where a participant gives succinct subjective information about their fatigue, in the form of a metaphor or non-generalizable feeling or experience. (3) Any text where a participant gives an opinion or interpretation of their fatigue, especially in relation to their experiences of fatigue before cancer treatment.
**“Energy Crashes”:** Descriptions of precipitous, sudden, uncontrollable, and relatively unpredictable losses of energy that require an immediate cessation of all action.	*“(Fatigue) comes out of nowhere. It is prolonged….it’s a crash. It’s an energy crash. It hits you upside the head. You can’t necessarily foresee… you try to hedge it. You try to measure what you do. Which is debilitating in itself. But you have to look at life a different way. And it’s different now because now I have to think about energy.*” (Participant 10) *“The fatigue will come over like a fog coming in on a Mountain Top. I’ve seen it happen. Come in and all of a sudden the snow drops. It comes in like a wall and then boom it hits. And you just have to lie down.”* (Participant 12) *“When I hit my max that’s when it’s just like the whole thing starts to…it’s like umm like you know when you take a sleeping pill and all of a sudden you start feeling like okay here it comes and you’re alert and aware that your body’s just beginning to sink? That’s the feeling that happens, it’s like, okay, here it comes and you just feel like [sighs] that’s it I’m done.”* (Participant 3)
**Ever-Present imbalance:** Descriptions of a chronic reduction in energy and the collateral effects this reduction has on a person’s sense of their own body and their ease in moving through the world.	*“I know that I physically feel like I can’t move…I feel like concrete…when I get tired the worst thing is cognitive, that I can’t think that’s really exhausting to me. And then I just get all flustered and I start crying.”* (Participant 7) *“It just feels like my whole body is tired, like the way that I’d describe it is that I cannot hold my own body up.”* (Participant 5) *“I don’t know, I kind of feel like my life is a little bit less than it used to be.” (Participant 2)*

**Table 3B**. **Interpersonal Influences**: **Clinical and Familial**	**Code Definition**: **1) Any text where a participant makes a statement related to their relationships; professional, familial, or medical.** **2) Any text where a participant makes a statement on how input from another person has affected their experience of fatigue.** **3) Any text where a participant makes statements about the relationship between their fatigue and social norms.**

***Social Perceptions of Fatigue****:* Any descriptions of how individuals in the participants’ extended social circle have interacted with their fatigue, and how those interactions made the participant feel.	*“It’s hard to connect with people because they think your either way down here, it’s like you’re developmentally disabled, or its like you don’t understand it…its, its so hard to, and its nobody’s fault, it’s really hard, it’s a very insidious thing, and that’s how exhaustion is. It’s a very insidious thing.”* (Participant 7) *“I think that the minute my hair grew back, and I looked like I could walk around, everyone thought I was fine, and that’s the end of it.”* (Participant 3) *“It’s hard because I look fine now, you know? I look fine. And ummm, people can’t understand, well family members are always the worst about these kinds of things, but family members can’t understand like ‘why can’t you do this, why can’t you do that, you’re fine, you don’t have cancer anymore’.” [laughs]* (Participant 4)
	*“If you go through it with somebody you love, its great…A guy has to have a bigger spine than a female going through cancer, simply because they’re going to take on the burden of not only their emotions, but hers as well. And you have to have a bigger spine. And my boyfriend has that.”* (Participant 6)
***Social Components of Medical Systems:*** any excerpt where a participant discusses their views, relationship, or experience with a doctor, hospital, or alternative treatment purveyor as it relates to their post-cancer related fatigue.	*“I was definitely feeling more lethargic…and I did mention it to my doctor, and it was a mix of empathy and non empathy.”* (Participant 1) *“It’s just not a topic that comes up. Not yet anyway. It’s kind of like when you’re leaving the heat of battle, they’re concerned with other things. The next surgery, the last surgery, how are you healing, is there any infection, they wanted to deliver the results to you and talk about the results and potential drugs you maybe should be taking. So they’re so busy with those things, just don’t get into that fatigue stuff.”* (Participant 13) *“yes, all my doctors have been great, I feel like I can tell them anything about what I’m going through and they understand and they listen and are receptive, they’ve never discounted any of my symptoms. They’re respectful of what I’m going through.”* (Participant 2)
***Cultural Values and Fatigue:*** *Descriptions of Participants’ experience with internalized social values related to energy levels and how these internalized values made them feel.*	*“I think fatigue in general is sort of…it umm, it feels embarrassing, I don’t know if it’s our culture, or my particular upbringing that you know…productivity and stamina and energy and a certain kind of energy is encouraged.”* (Participant 1) *“I guess it’s this culture we’ve inherited about the, you know the work ethic and all that sort of thing…Every slot filled.”* (Participant 7) *“Mcain’s had cancer it’s not part of his identity it affected his experience and then it was done, right? But for women I think it can be different, right?”* (Participant 5)

**Table 3C. Changes in Bodily Awareness Following Cancer Treatment**	**Code Definition:** **(1) Any portion of the text where the participant describes a change in the quality of or their capacity for bodily awareness**

***Experiences of Anxiety:*** *Any description of anxiety that occurs as a function of cancer treatment. 2) Any description of how a participant’s awareness of their body has changed due to chronic anxious monitoring of bodily sensations.*	*“I sit and my knees hurt or my legs hurt, and I say, ‘uh oh I hope it’s not cancer’, you know it’s just the constant level– like there’s a low level of anxiety all the time that I think contributes, and it makes you much more aware of what’s going on inside your body.”* (Participant 3) *“I kept a chap stick in my bra, and so I had a chap stick tube right here, the big one would hold it in, and I would go like this, (touches chest) being like “Oh no I have a tumor,” and would freak out that I had cancer again, but it was just my chap stick, so there’s like that level of false positives.”* (Participant 5) *“Every time I get an ouch or an ache, I’m like ok what now.”* (Participant 6)
***Shifts in Bodily awareness as a function of Fatigue:*** *Any description of a shift in the resting state perception of bodily sensations. 2) Any description of actions a participant takes with this newfound perceptual capacity.*	*“If it really becomes overwhelming then I listen to my body and if I feel like my body is telling me to take some time out to stop what I’m doing then I do, I feel like I’ve become more respectful of my body signals since all this happened.”* (Participant 2) *“I learned how to access that wisdom in my body. When It’s not the little thinking brain…what the cancer has brought me, the diagnosis and the dreams and everything,, brought me to knowing how to* *access that.*” (Participant 11) *“I think it emanates from within, because I’m able now to, I’m out of the I’m out of the wheel going round and round. You know? I um I’m off that track and I’m able to, I feel like I get as I recognize that that’s not the only value is doing those things physically. I think I get satisfaction from that. I think that emanates from within.”* (Participant 7)***Practices that help with fatigue:*** *Any description of a practice described as helping to ameliorate a participants’ experience of fatigue.*	*“Yeah. My personal… I like to call it my toolbox, the tool that I’m using with this crisis in my life is actually music and meditative music, and worship…That I find helps to bring me to another level where I can move forward and feel better.”* (Participant 12) *“Doing the dance stuff, and more so then running, you know, because there’s something that sort of carries you away within it, you sort of surrender to it as opposed to running which is so repetitive. And you know, I like, it’ll alleviate it while I’m there, I’ll be like wow I was so tired when I came in but I’m getting kind of energized from this and I’ll feel good for a while afterwards.”* (Participant 1) *“I think yoga has helped me that way because I realize how the whole body is connected from your toes to the top of your head and if your walking it’s doing good for not just your legs but your arteries your veins, your heart, your muscles…your brain. Whatever kind of exercise your doing it’s doing something for all of those areas.. no matter what exercise your doing it can work for all of those areas.”* (Participant 2).
	

### Changes in Attention Toward Bodily Sensations Following Cancer Treatment

The means by which CRF manifested in the felt sense of the body was highly heterogeneous for our participants. For those who felt their fatigue could be linked to a specific body location, CRF was almost always centered on their head. The vast majority, however, had trouble pinning CRF to an explicit part of the body as one could with muscular weakness or pain.

*“I think it’s more of a sensation. I don’t think it’s from my body or my mind per se, it’s just I get, I get to the point where it’s like, maybe I tried to stay up. And sometimes it works and sometimes it doesn’t. I don’t know maybe it could be like one of those you know the internal clock? Maybe I have to reset my internal clock”* (Participant 6).

For these participants, fatigue appeared qualitatively to be akin to a loss of communication across the body, leaving only an overwhelming need to cease activity. This lack of meaningful communication throughout the body was contextualized for participants by the trauma of their cancer experience. For many, the experience of fatigue was fundamentally connected to the violence of being treated for cancer.

“I think on some fundamental level my body has no idea what f^*****^g happened to it, it was just existing and trying to be nice, and then all of a sudden comes the poison and the cutting, ya know, cutting parts of me out and the medication and I think my body, sort of at a pseudo existential level is completely disoriented and my bits. my bits have no idea what’s going on, you know, and I haven’t even recovered (Participant 5).

The most consistent and tangible way this trauma appeared to manifest for our participants was in their articulated fear of recurrence. Given that the object of anxiety, a tumor, manifests within the body, the interior space becomes a staging ground for the harrowing “assault” of treatment, which was also a harbinger of fatigue. This affective dimension of post-cancer experience manifests as a shift in the valence of interoceptive and somatic afferents. Because of the constant drive to interrogate sensations as they arose, participants commonly reported a chronic pull toward body-centered rumination.

*I would want to be aware and try to catch things, if anything is going to happen I ‘d want to try to catch it as early as possible, that’s why I’m paying more. doing a little more physical naval gazing,(laughs) than I would, you know I would normally have done before* (Participant 4).

These shifts toward an anxiety-driven monitoring of bodily sensations represents a pathological face of one of the most surprising consistencies that emerged from our participant’s accounts of CRF. Many of our participants reported, contrary to our expectations, a much greater awareness of their bodily sensations on a day-to-day basis.

*“I think fatigue is real and I think when it happens it’s part of your mind-body mechanism. And it’s good to honor fatigue when you can*… *I think it’s part of respecting your body and not always being so focused on what’s going on in your mind. Just stopping, like stopping thinking about when it happens and just like tune in to what’s happening in your body, and I’ve been able to do that more since I’ve had my breast cancer. I don’t know maybe you need to go through a life changing event like that to become more honorable toward your whole self really, not just the mind but the body as well, they’re both interconnected as far as I’m concerned, it’s all one” (Participant 3).*

This increase in attention toward bodily sensations appeared to be driven directly by the fatigue itself and an understanding that, “honoring the fatigue,” (Participant 3) was an effective approach for buffering the “crash” of fatigue and potentially ameliorating symptomology. The relationship between the shifts in bodily attention driven by anxiety and those driven by a desire to, “honor fatigue,” often coincided dynamically over the course of the day. Many of our Participants described a newly developed metacognitive capacity that allowed them to be aware of bodily signals without necessarily ruminating on their specific characteristics.

*“So I spent a lot of time driving myself crazy by trying to categorize the symptoms- label their intensity, figure out what caused them, come up with a treatment plan, but when they’re subclinical I can’t do it*… *and sometimes I get to this loop where I’m trying to like interpret my symptoms but I have to be like no I don’t have enough data, all I can do is honor the limitation I feel right now” (5).*

Practices that worked to either actively or passively facilitate shifts in non-judgmental embodied awareness were often highlighted by Participants as being particularly helpful tools for moving through the experience of fatigue. The nature of these practices was varied, examples being; visualization meditations (Participants 5 and 3), movement based practices like yoga (Participants 2 and 4), dance (Participant 1), Ecstatic Christian prayer (Participant 12), shamanic ritual (Participant 11), and gardening (Participant 7). The shared faculty of all these practices was the apparent foregrounding of the body during an experience without it being an intentional object of focus; the experience of losing oneself in an action. Our analysis revealed that one of the most important factors in the experience of CRF was the change in our participant’s attention toward, and interpretation of, their bodily signals. This changed experience of the body was not apparently linked to any specific practice, social climate, or personal belief system, but rather arose as a result of learning to, “honor the limitation,” that followed from living with chronic fatigue. In addition to this apparently beneficial mode of awareness, there was also a more pernicious anxiety-driven shift in sensory cognizance, such that innocuous bodily signals or pain took on a different valence and were thus amplified by attention. For many of our participants exercises that facilitated a shift in the experience of the body were particularly effective at changing a participant’s outlook on post-cancer circumstances and in some cases ameliorating CRF altogether.

## Discussion

In this paper our objective was to understand how social, emotional, and bodily experiences coalesced in the fatigue experience of Cancer Survivors. To this end we utilized team-based qualitative analysis to illuminate trends in the subjective experience of CRF. Our analysis revealed that CRF was defined by two broad elements. One was the sudden, overwhelming, exhaustion one participant termed an “energy crash.” The other was a subtler, chronic, shift in the experience of the body that led to a sense of perpetual heaviness. Interestingly, other groups have documented similar dimensions of fatigue. For example, Söderberg et al. (2002) in their analysis of the experiences of fatigue in individuals with fibromyalgia describe a two faced chronic fatigue that is akin both to, “walking in the fog or being enveloped in wool,” as well as, “being stunned or anesthetized.” Likewise, for fatigued individuals with chronic pulmonary disease fatigue could, “(occur) slowly and gradually” or, “come on fast” (Stridsman et al., 2014), and for fatigued patients with multiple sclerosis the body was experienced as being, “numbed, dead, and not quite awake,” and fatigue was, “a form of paralysis…that could not be stopped in any way” (Flensner et al., 2003).

The dynamic interplay between interpersonal relationships, perceived moral implications of low energy states, and the disease experience of chronic fatigue has been well documented in CRF (Holley, 2000; Oktay et al., 2011) as well as a number of other chronic conditions associated with fatigue (Ware, 1992; Arroll and Howard, 2013; McManimen et al., 2018). In our study we found that social factors often led participants to dismiss their experiences of fatigue, viewing exhaustion as being reflective of personal or psychological weakness instead of a biological phenomena beyond their control. This tendency changed the means by which CRF was experienced by participants and shifted the reference frame of fatigue from a focus on internal state to one based around productivity. For some women, embracing this consummate of fatigue was effective at helping them to structure a sense of well-being and return to normalcy (Participants 9 and 6). For others, these social factors effectively obscured CRF such that for years following treatment some had no comprehension they were chronically exhausted, only that they were, “screwing everything up”(Participant 7). As such, the well established notion that some individuals are capable of “mastering their fatigue” was seen to hold true in a few select cases, but for the majority of participants did nothing but shackle their attempts to find peace and normalcy in the face of their fatigue.

Coupled with this sense of guilt and inadequacy was another emotional component of the post-cancer experience, fear of recurrence. Both fear of recurrence and the related tendency to catastrophize have been directly related to the severity of CRF (Jacobsen et al., 2004; Young and White, 2006). For our participants fear of recurrence existed directly in relation to the trauma of their cancer treatment, and indeed the extent of their current fatigued circumstances.

Fear of Recurrence and the internalization of socially subsidized views of productivity interacted dynamically with one of the primary themes to emerge through our analysis, a reported shift in the level and quality of bodily attention in fatigued cancer survivors. This seemingly counterintuitive change in interoceptive awareness has likewise been reported for chronic fatigue patients and is indeed often described as being a functional component of the disorder (White, 2004; Jones, 2008; Kadota et al., 2010).

For the women in our study moving through the world was no longer an unconscious process. As a function of their exhaustion they were forced to devote cognitive resources to previously unconsidered actions. The need to chronically maintain an awareness of how bodily resources were being utilized is functionally akin to the rumination that accompanied fear of recurrence. Both factors constantly force a survivor’s awareness back into the body with an associated negative valence. The experience of being in a fatigued body was emotionally charged for many participants who felt guilty and insufficient as a result of their fatigue. As such, the constant pull back to the body as a necessary function of conserving energetic resources was also a constant return to socially subsidized feelings of laziness and failure. Likewise, the experience of constantly tracking bodily sensations drained cognitive resources and represented a significant source of emotional distress. Chronically being aware of the body can therefore be seen as both a symptom and a cause of the altered embodied awareness present in CRF.

In direct response to the constant convergent experience of anxiety and energetic depletion, many participants described being forced to more actively listen to their bodily sensations, this shift toward a state of increased interoceptive awareness facilitated behaviors that would be supportive and nourishing, rather than oriented around socially subsidized definitions of productivity.

Importantly, when participants described practices that were helpful in dealing with their fatigue, they often used language that denoted a departure from an explicit awareness of bodily sensations: activities that helped participants to feel more energized and tap into a deeper level of embodied understanding. In their paper on the shared ground of common mind body exercises Mehling et al. (2011) stat that,

*Body awareness-enhancing therapies resume an embodiment process that has been disrupted in its unfolding. These elements support the common goal of all practices, the integration of mind, body and life context* (Mehling et al., 2011).

It is possible that beyond the direct physiological benefits of something like yoga or dance there is also a shift in the quality of bodily attention carried by a practitioner into their, “life context.” If this is the case these assorted practices can be seen as behavioral interventions designed to change the tendency to meaningfully tap into the signals that arise from the body rather than simply physical exercises. Indeed recent evidence from a number of sources supports this notion (Kerr, 2002; Mustian et al., 2007; Bower et al., 2012; Thomas et al., 2014).

What emerges from this analysis is a view that the difference between ruminative and nourishing awareness of the body in cancer survivors is a matter of what heuristic is applied to the experience of fatigue. When an internal sensation, can be meaningfully understood as fatigue, it can lead to a set of nourishing actions that are squarely in line with the needs of the body. Alternatively if an individual interacts with bodily sensations on the basis of an embedded cognitive framework (fear of recurrence or a sense of guilt/responsibility) then the fatigue or pain that arises from the body is characterized as immoral or scary, and an individual continues to ruminate on the sensation without changing their bodily state.

### Limitations

Though this work presents preliminary evidence about the changed experience of the body in fatigued Breast cancer survivors, there are a number of serious limitations. First and foremost, although our study group came from a diverse socioeconomic background they were all of Caucasian descent. It is quite possible that the socially subsidized perceptions we describe in this manuscript are culturally specific and will not have a bearing on the experience of women from different ethnic and social backgrounds. Moreover, our study sample was small with a large inter-participant spread both in terms of the time since cancer treatment, the severity of experienced fatigue, and the form of cancer treatment. These factors may have limited the tractability of our analysis and lead to a more heterogeneous set of responses than might otherwise be expected.

### Future Directions

Future Studies should attempt to address the aforementioned issues by conducting cross-cultural analyses on the experience of CRF. Moreover, in this study we only examined the experience of Breast Cancer Survivors, future studies should compare and contrast the felt experience of individuals who have suffered from different forms of cancer.

The trends outlined in our study closely mirror a number of previous reports on the experiences of patients suffering from other, in large part, medically unexplained syndromes associated with chronic fatigue (Bennett et al., 2007; Kanaan et al., 2007; de Lourdes Drachler et al., 2009; McBeth et al., 2015; Rosendal et al., 2017; Petersen et al., 2020). These phenomenological similarities could result for a few different reasons:

(1) The shared cultural frame applied to disorders that elude treatment leads to parallel processes of disavowal, alienation, and erasure on the part of medical institutions and interpersonal relationships toward those who are suffering.
(2) Separate syndromes that result in chronic fatigue share a common set of etiological factors, which in turn lead to distinct disease states that are actually different faces of the same biological phenomena.
(3) Because describing the experience of having a chronic and medically undefined condition is so challenging, researchers are only reporting those aspects of a disorder that can be easily disentangled. Thus the apparent consistencies between disorders are actually a function of the coding process wherein similarities between experiences are emphasized over the inherent heterogeneity of qualitative data.

Put more simply, the aforementioned similarities between different conditions could be the result of cultural, biological, or methodological factors. Future studies should carefully evaluate the shared elements between different syndromes resulting in chronic fatigue in order to create a cogent and clinically tractable model of their interactions.

### Conclusion

In this manuscript we put forward an analysis of the complex and detrimental experience of Cancer Related Fatigue. Fatigue existed on multiple time scales for our participants who reported both sudden, “energy crashes,” and a more difficult to define chronic experience of imbalance. We found that very often women reported feelings of guilt and unworthiness as a function of their fatigue. In accounting for this chronic fatigue many women reported becoming more aware of their bodily sensations. This new sense for the body existed both as a pathological fear of recurrence, and a means of taking actions that were squarely in line with the needs of the body.

Clinical practitioners should carefully consider the dynamic interplay of these factors when offering advice and insight on what can be expected from post-cancer experience. If we are to truly answer the ongoing, “silent cry” of cancer survivors it will require an attentive foregrounding of their experiences. The shifts in bodily attention described by our participants may represent an important and previously underdeveloped aspect of the disorder that can be targeted in clinical interventions moving forward.

## Data Availability Statement

The datasets generated for this study are available on request to the corresponding author.

## Ethics Statement

The studies involving human participants were reviewed and approved by Brown University Research Protections Office. The patients/participants provided their written informed consent to participate in this study. Written informed consent was obtained from the individual(s) for the publication of any potentially identifiable images or data included in this manuscript.

## Author Contributions

CP undertook the interviews and completed the initial codebook. CZ and CP drafted the manuscript. CK brought forward the initial idea and secured funding. TK and LC helped finalize the codebook and offered extensive edits on the manuscript. All authors contributed to the article and approved the submitted version.

## Conflict of Interest

The authors declare that the research was conducted in the absence of any commercial or financial relationships that could be construed as a potential conflict of interest.
